# Machine learning-based prediction of hernia risk in peritoneal dialysis patients: a comparative study of models and SHAP-driven interpretability analysis

**DOI:** 10.3389/fmed.2026.1687055

**Published:** 2026-03-04

**Authors:** Yugang Cao, Xun Hu, Tao Fang, Jun Guo

**Affiliations:** Department of Hepatobiliary Surgery, Huangshi Central Hospital, Hubei Key Laboratory for Kidney Disease Pathogenesis and Intervention, Hubei Polytechnic University School of Medicine, Huangshi, Hubei, China

**Keywords:** end-stage renal disease, hernia risk prediction, machine learning, peritoneal dialysis, SHAP analysis

## Abstract

**Purpose:**

This study aimed to predict the hernia risk in peritoneal dialysis patients using machine learning (ML) models and conduct an interpretability analysis.

**Methods:**

A total of 1,144 eligible PD patients (2010–2024) were divided into training (*n* = 800) and external validation (*n* = 344) cohorts. Nine ML models were constructed, and SHAP analysis was used for interpretability. Model performance was evaluated via AUC, accuracy, DCA, etc. An online visualization tool based on the optimal model was developed using R Shiny and deployed for clinical use.

**Results:**

The Random Forest (RF) model performed optimally (training AUC = 97.99%, validation AUC = 93.66%), identifying 9 core risk factors (age, BMI, PDV, albumin, smoking history, history of abdominal surgery, high peritoneal transporter status, COPD, and CAPD modality). SHAP clarified non-linear effects of these factors. The developed R Shiny-based online tool (https://caoyugang.shinyapps.io/appforpub/) enables real-time risk calculation through intuitive input of clinical indicators, providing risk stratification and personalized clinical recommendations.

**Conclusion:**

The RF model achieves high-accuracy and interpretable hernia risk prediction in PD patients. The R Shiny-based online tool facilitates clinical risk stratification and early intervention, improving patient prognosis.

## 1 Introduction

ESRD constitutes a severe global public health challenge. PD serves as a vital renal replacement therapy that sustains the lives of millions of ESRD patients worldwide (1). PD offers distinct advantages, such as enabling home-based treatment and preserving residual renal function. However, it is accompanied by unique complications, among which abdominal wall hernia is particularly prevalent and clinically impactful (2). Epidemiological studies have reported that the incidence of hernia in PD patients ranges from 10 to 30% (3), and recent data from a 2024 multi-center cohort study further confirmed that hernia-related complications increase PD technique failure risk by 2.3-fold and in-hospital mortality by 1.8-fold. Once a hernia occurs, it often leads to adverse outcomes including PD technique failure, patient hospitalization, and even increased mortality risk—primarily due to life-threatening complications like hernia incarceration or strangulation. Therefore, the timely identification of PD patients at high risk of hernia is crucial for optimizing clinical management strategies and improving patient prognosis.

Traditional statistical models, such as logistic regression (LR), have long been applied to explore hernia risk factors in PD patients. One study used LR to analyze hernia risk and identified only three risk factors: age, albumin level, and prior abdominal surgery. A notable limitation of this LR-based study was its failure to capture the non-linear relationship between PDV and hernia risk—specifically, the sharp increase in hernia risk when PDV exceeds 5 years, which is a key clinical observation. Such limitations stem from the inherent constraints of traditional models, including their reliance on rigid assumptions (e.g., linearity between predictors and outcomes) and their inability to detect complex non-linear interactions among variables. These shortcomings highlight the need for more advanced analytical tools to address the complexity of hernia risk prediction in PD patients.

In recent years, machine learning (ML) algorithms have drawn growing attention in clinical risk prediction, thanks to their strong ability to handle high-dimensional data and capture non-linear relationships. Relevant systematic reviews have indicated that tree-based ensemble models (such as random forest and gradient boosting) tend to perform better than traditional models in predicting PD-related complications (4). However, existing research on hernia risk prediction in PD patients still has notable limitations. First, most studies focus on a single ML model, lacking systematic comparisons across multiple algorithms to identify the optimal predictive tool—some comparative studies even fail to include key tree-based models, which restricts the ability to determine which algorithm can best balance accuracy and generalization (5–7). Second, although some studies have integrated multiple clinical variables to construct prediction models, they often overlook PD-specific indicators that are later verified as critical predictive factors (8). Third, few studies incorporate interpretive frameworks; relevant meta-analyses have pointed out that explainable methods are rarely used in ML-based PD complication research, which hinders clinical trust and adoption (9). Even studies that apply interpretive tools often do not combine feature selection methods to screen core variables, leading to potential redundancy in predictive factors and reducing the reliability of identifying risk factors (10). Additionally, while recent research has emphasized the importance of external validation for ensuring model generalizability, most existing hernia prediction models lack independent validation cohorts (11).

ML algorithms, including RF and Light Gradient Boosting Machine (LightGBM), offer significant advantages over traditional models. They excel at handling high-dimensional data, identifying hidden predictive patterns, and capturing non-linear relationships and interactions between variables—making them promising tools for clinical risk stratification. Despite this potential, there remains a scarcity of comparative studies that evaluate multiple ML models for hernia risk prediction in PD patients. Furthermore, few studies have integrated interpretive frameworks (e.g., SHAP analysis) to enhance the clinical trustworthiness and applicability of model outputs, which is a critical barrier to the translation of ML models into clinical practice.

Against this backdrop, the present study aimed to address these research gaps with three key objectives: (1) Develop and systematically compare the performance of nine ML models in predicting the risk of abdominal wall hernia in PD patients with ESRD; (2) Identify key predictive factors for hernia—specifically, Least Absolute Shrinkage and Selection Operator (LASSO) regression will be used for dimensionality reduction to screen core variables from high-dimensional clinical data, while SHAP (SHapley Additive exPlanations) analysis will be employed to interpret the non-linear effects of these variables on hernia risk; the combination of LASSO and SHAP will enhance the reliability and interpretability of the identified risk factors; (3) Validate the optimal ML model using an external cohort to ensure its generalizability across different clinical settings. By achieving these objectives, this study seeks to provide a robust, interpretable predictive tool that can support clinicians in proactively managing hernia risk in PD patients.

## 2 Materials and methods

### 2.1 Study subjects

#### 2.1.1 Data sources

The study data were derived from patients with end-stage renal disease (ESRD) who received peritoneal dialysis (PD) treatment at Huangshi Central Hospital and its Puai Campus between October 2010 and October 2024. Data for the training cohort were obtained from PD patients at the main campus of Huangshi Central Hospital, while data for the external validation cohort were collected from PD patients at Puai Campus during the same period. The research team retrospectively extracted patient data through the hospital’s electronic medical record system, and all cases of abdominal wall hernia were confirmed by imaging examinations such as computed tomography (CT), magnetic resonance imaging (MRI), or B-mode ultrasound.

#### 2.1.2 Inclusion and exclusion criteria

To ensure the homogeneity of the study population and the validity of outcome indicators (focusing on new-onset abdominal wall hernia during PD), strict inclusion and exclusion criteria were formulated:

Inclusion criteria:

1. Aged 18–80 years, excluding pediatric patients and elderly individuals with excessively high competing risks of death or non-PD-related complications that could confound hernia risk analysis;
2. No prior diagnosis of abdominal wall hernia or intra-abdominal hernia before the initiation of PD. This criterion is critical to clarify the association between PD-specific factors (such as sustained intra-abdominal pressure elevation and chronic peritoneal inflammation) and new-onset hernia, avoiding confounding results by patients with impaired baseline abdominal wall integrity.

Exclusion criteria:

1. Hernia unrelated to the PD period (e.g., undocumented hernia before PD initiation, or hernia caused by trauma, abdominal malignancy, or other non-PD factors), ensuring the outcome reflects PD-related pathogenesis;
2. Previous hernia repair surgery. Patients who underwent hernia repair have altered abdominal wall anatomical structures (e.g., mesh implantation, fascial suturing), and their recurrence risk factors are essentially different from those with intact abdominal walls;
3. Follow-up duration < 6 months or missing key variables (such as PD vintage, serum albumin, peritoneal transport status), avoiding missed outcome events or data defects in model construction;
4. Pregnancy during PD. Pregnancy can independently increase intra-abdominal pressure and alter abdominal wall biomechanics, introducing irrelevant confounding factors;
5. Abdominal wall trauma during PD. Trauma directly causes abdominal wall defects, with a pathogenic mechanism distinct from PD-induced chronic pressure or inflammation.

#### 2.1.3 Supplementary specifications for laparoscopic PD catheter placement

For patients undergoing laparoscopic PD catheter placement, a standardized intraoperative assessment protocol for subclinical abdominal wall hernia defects was implemented:

Exploration scope: The surgical team systematically inspected the entire abdominal wall, focusing on high-risk areas for herniation such as the umbilical region, inguinal canal, previous abdominal surgical incisions, and epigastric area;
Verification method: Under laparoscopic visualization, the team checked for fascial defects, protrusion of intra-abdominal contents, or weakened tissue consistency. If an asymptomatic subclinical hernia (defined as a fascial defect ≥ 1 cm without clinical manifestations before surgery) was identified, the patient was directly excluded to ensure all subsequent hernia events in enrolled patients were new-onset during PD.

#### 2.1.4 Cohort grouping

**Figure 1:**
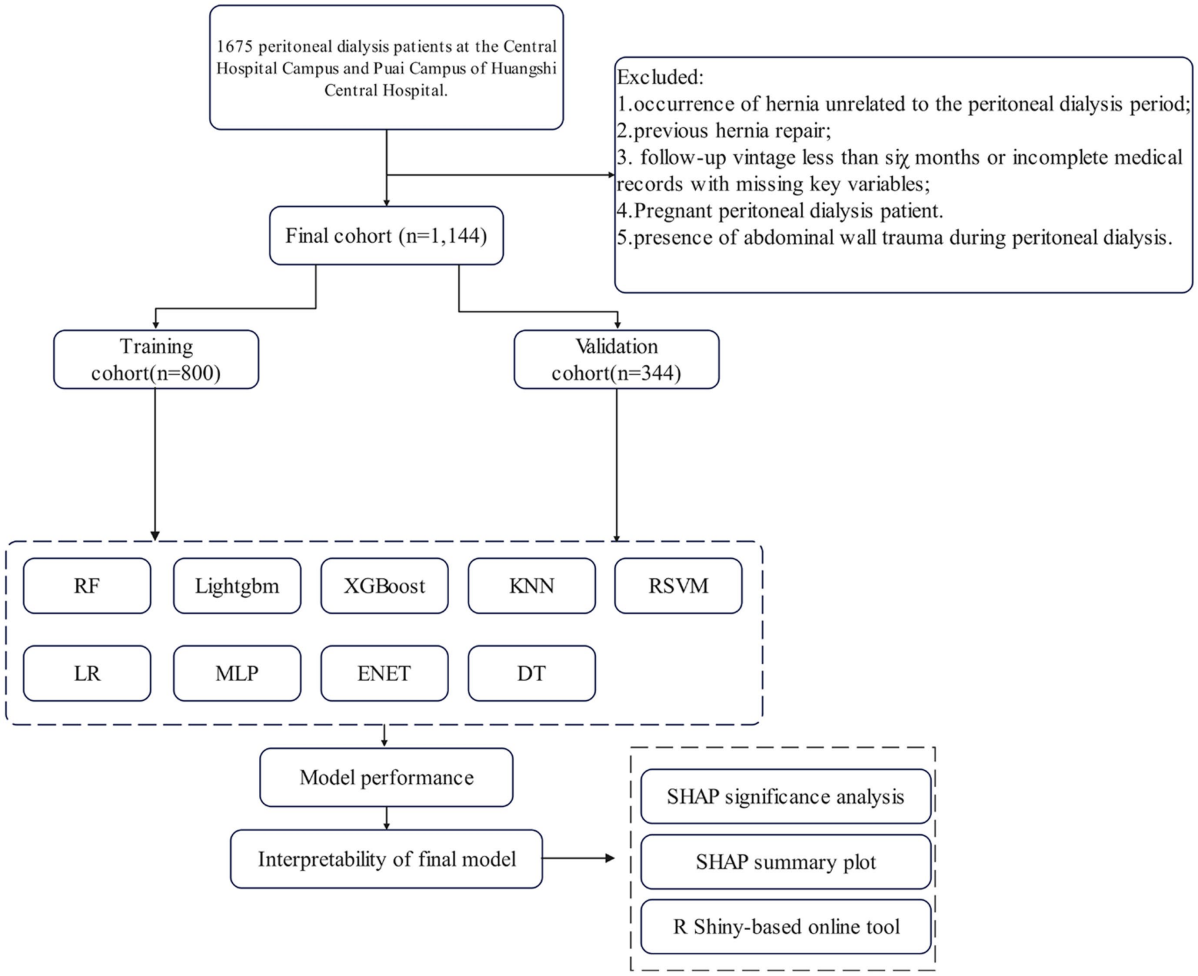
Flowchart outlining patient’s enrollment and study design.

After screening based on the above criteria, a total of 1,144 eligible patients with renal insufficiency were finally enrolled, and randomly divided into a training cohort (*n* = 800) and an external validation cohort (*n* = 344) in a 7:3 ratio. It should be specifically noted that patients with a history of non-hernia abdominal surgery (e.g., gastrointestinal surgery, hepatobiliary surgery) were not excluded, and this history was retained as a potential predictive variable—such surgeries may cause residual abdominal wall damage (e.g., fascial scarring), thereby increasing the risk of PD-related hernia. Unlike targeted intervention for hernia repair surgery, it is a natural risk factor. The patient enrollment process and study design are shown in Figure 1.

### 2.2 Data collection

#### 2.2.1 Definition and diagnostic criteria of hernia

PD-related hernia was defined as a protruding mass formed when intra-abdominal organs or tissues push through a weak spot, defect, or opening in the abdominal wall due to persistently or intermittently elevated intra-abdominal pressure during PD. This includes inguinal hernias, umbilical hernias, incisional hernias, epigastric hernias, and other rare types such as lumbar hernias.

Diagnosis required meeting both: ① Clinical physical examination identifying reducible or irreducible bulges; ② Imaging examinations (ultrasound or CT) confirming the location, size, and contents of the hernia, excluding other abdominal masses, and determining the presence of complications such as incarceration or strangulation. Subclinical hernias (asymptomatic but with abdominal wall defects and protrusion of intra-abdominal contents indicated by imaging) were also included in the analysis. In addition, the time of hernia occurrence (interval from PD initiation to first diagnosis), anatomical location, clinical symptoms, and treatment methods were recorded to ensure data completeness.

#### 2.2.2 Sociodemographic and behavioral characteristics

Sociodemographic information including age, gender, marital status (married/unmarried), place of residence (urban/rural), and years of education was collected; regarding behavioral characteristics, smoking history was recorded as a binary variable (yes/no), and physical activity intensity was assessed using Metabolic Equivalent of Task (METs)—based on the International Physical Activity Questionnaire, activity intensity was classified into four levels: light effort (< 3.0 METs), moderate effort (3.0–6.0 METs), vigorous effort (6.0–8.0 METs), and very vigorous effort (> 8.0 METs). Among them, 1 MET was defined as the energy expenditure of a healthy adult at rest while sitting quietly (approximately 3.5 mL O₂/kg·min).

#### 2.2.3 Health status-related indicators

Based on previous studies and clinical experience, health indicators potentially associated with hernia risk were included:

1. History of chronic diseases: Including diabetes mellitus, heart disease, chronic obstructive pulmonary disease (COPD), connective tissue disorders (CTDs), etc.;
2. Prior abdominal surgery history: Referring to various abdominal surgeries performed before PD initiation (excluding PD catheter placement itself), including hernia repair surgery before PD;
3. Anthropometric indicator: Body mass index (BMI);
4. PD-specific indicators: Daily ultrafiltration volume (DUV), serum creatinine, urea clearance index (Kt/V), peritoneal equilibration test (PET) results, PD modality (continuous ambulatory peritoneal dialysis [CAPD]/automated peritoneal dialysis [APD]), etc.

#### 2.2.4 Definition and assessment methods of connective tissue disorders

Definition: CTDs refer to a group of autoimmune or inflammatory diseases characterized by pathological involvement of connective tissues (such as collagen, elastin, and proteoglycans) throughout the body. These diseases often affect multiple organ systems, and their relevance to this study lies in their potential to impair abdominal wall integrity—for example, through inflammation-induced weakening of fascial collagen, vascular dysfunction reducing tissue repair capacity, or direct structural damage to muscle-fascial layers. Common CTDs included in this study were systemic lupus erythematosus (SLE), rheumatoid arthritis (RA), systemic sclerosis (SSc), Sjogren’s syndrome (SS), polymyositis/dermatomyositis (PM/DM), and antiphospholipid syndrome (APS).

Assessment methods: A combination of clinical, laboratory, and imaging evaluations was used to ensure diagnostic consistency:

1. Clinical diagnosis: Patients were identified as having a CTD only if they met the specific disease classification criteria established by the American College of Rheumatology (ACR) or the European League Against Rheumatism (EULAR) (e.g., SLE required meeting ≥ 4 of 11 criteria in the 2019 EULAR/ACR Classification Criteria);
2. Laboratory verification: Routine screening for antinuclear antibodies (ANA) was performed via indirect immunofluorescence, followed by specific antibody testing for disease subtyping (e.g., anti-double-stranded DNA [dsDNA] antibodies for SLE subtyping). Meanwhile, high-sensitivity C-reactive protein (hs-CRP) and erythrocyte sedimentation rate (ESR) were measured to assess disease activity;
3. Imaging and functional evaluation: For patients with suspected organ involvement, additional examinations such as chest CT (to evaluate SSc-related interstitial lung disease) and electromyography (EMG) (to evaluate PM/DM-related myositis) were performed. Only patients who met both clinical criteria and laboratory/imaging evidence were classified as “CTD-positive,” while those with isolated autoimmune antibody positivity (without clinical symptoms or failing to meet classification criteria) were excluded.

### 2.3 Follow-up

With the date of PD catheter placement as the starting point of follow-up, a standardized follow-up plan was formulated to ensure systematic monitoring of PD-related complications (focusing on abdominal wall hernia) and accurate capture of outcome events.

#### 2.3.1 Follow-up schedule and modalities

A combination of in-hospital outpatient follow-up and telephone follow-up was adopted (telephone follow-up was used for patients unable to attend in-person due to geographical or physical constraints). The follow-up frequency was stratified by the time since catheter placement:

First follow-up: Scheduled 1 month after hospital discharge, primarily to assess PD catheter patency, resolve early operational issues, and collect baseline laboratory and imaging data;
Within 1 year after catheter placement: In-hospital follow-up once every 2 months to closely monitor dynamic changes in abdominal wall integrity and PD-specific indicators. All patients underwent a unified PET examination 3 months after the actual initiation of PD therapy (date of the first dialysate exchange) to avoid interference from early post-catheterization peritoneal inflammation or catheter irritation, ensuring the results reflect stable peritoneal transport status (low, low-average, high-average, or high transporter status);
After 1 year of catheter placement: In-hospital follow-up once every 3 months. Patients initially classified as high or high-average transporters underwent repeated PET examinations annually to capture the impact of long-term changes in peritoneal transport function on hernia risk.

The median follow-up duration of the entire cohort was 65 months. If patients discontinued PD treatment (e.g., switched to hemodialysis), died, or were lost to follow-up, the last valid follow-up time was taken as the censoring time point. Peritoneal dialysis vintage (PDV) was defined as the time interval from the date of PD catheter placement to the end of follow-up or the date of hernia occurrence.

#### 2.3.2 Follow-up evaluation content and outcome definition

Each in-hospital follow-up included the following comprehensive assessments:

Laboratory examinations: Venous blood samples were collected to measure serum creatinine (to evaluate residual renal function) and serum albumin (a key marker of nutritional status and abdominal wall integrity, with low albumin associated with increased hernia risk);
Imaging examinations: Routine abdominal CT scans were performed, which had higher sensitivity in detecting subclinical hernias compared to ultrasound;
Clinical physical examination: A dedicated abdominal surgeon performed systematic abdominal palpation, focusing on common hernia sites such as the umbilical region, inguinal canal, and incisional areas, to determine the reducibility of bulges and assess signs of hernia complications (e.g., tenderness indicating incarceration).

The primary outcome of the study—"time to abdominal wall hernia”—was defined as the interval from the date of PD catheter placement to the first confirmed diagnosis of abdominal wall hernia. The diagnosis time of subclinical hernia was based on the date of the CT scan that first identified the abdominal wall defect.

### 2.4 Statistical analysis

All statistical analyses were performed using R software (version 4.5.1), with a two-sided *p* < 0.05 considered statistically significant.

#### 2.4.1 Descriptive statistics and sample grouping

Continuous variables were expressed as mean ± standard deviation, and group comparisons were performed using univariate analysis; categorical variables were expressed as frequency (proportion), and group comparisons were performed using the chi-square test or Fisher’s exact test (for subgroups with small sample sizes);

The cohort was randomly divided into a training set (*n* = 800) and a validation set (*n* = 344) in a 7:3 ratio using R’s built-in random number generator (seed value: 1234). The training set was used for model construction, and the validation set was used for external validation.

#### 2.4.2 Feature selection

After all models were constructed and the optimal model (random forest [RF] model) was determined, feature importance selection was performed based on this optimal model: SHapley Additive exPlanations (SHAP) analysis was used to quantify the mean absolute SHAP value of each candidate variable, and variables were sorted in descending order of this value. The 9 core variables that contributed most significantly to hernia risk prediction were selected, namely age, BMI, PD vintage (PDV), serum albumin, smoking history, history of abdominal surgery, high peritoneal transporter status, COPD, and CAPD modality. Post-selection verification confirmed that each core variable corresponded to ≥10 hernia outcome events, fully complying with the EVP principle and avoiding overfitting caused by insufficient event-variable matching.

Furthermore, Spearman rank correlation analysis was used to verify the correlation between continuous variables. The results showed that the absolute values of all variable correlation coefficients were ≤ 0.15 (all *p* > 0.05), with no obvious multicollinearity. No additional dimensionality reduction processing was required, ensuring the stable estimation of the independent effect of each variable on the outcome while maintaining the parsimony of the model — a key complement to the EVP principle in optimizing prediction model performance.

#### 2.4.3 Model construction and optimization

Based on the selected core variables, 9 machine learning (ML) algorithm models were constructed: logistic regression (LR), decision tree (DT), elastic net (ENet), extreme gradient boosting (XGBoost), k-nearest neighbors (KNN), light gradient boosting machine (LightGBM), multilayer perceptron (MLP), regularized support vector machine (RSVM), and random forest (RF).

Model stability guarantee: Hyperparameter optimization was performed through 5-fold cross-validation and grid search (see Supplementary materials);

Model calibration: Calibration curves were used to assess the consistency between predicted probabilities and actual hernia incidence, and the Brier score was used to quantify calibration errors (lower scores indicate better calibration);

Threshold selection: The optimal cutoff value was determined based on the receiver operating characteristic (ROC) curve to balance model sensitivity and specificity.

#### 2.4.4 Model performance evaluation

Multi-dimensional indicators were used to evaluate model performance:

Discriminative ability: Area under the ROC curve (AUC), accuracy, sensitivity, specificity, recall, and F-measure;
Clinical benefit: Decision curve analysis (DCA) was used to evaluate the net clinical benefit of the model;
Optimal model selection: The optimal model was determined based on the highest AUC and accuracy in the validation set.

#### 2.4.5 Model interpretability analysis

SHapley Additive exPlanations (SHAP) analysis was used to interpret the optimal model:

Feature importance: The contribution of each variable was quantified through SHAP summary plots and feature importance ranking (mean absolute SHAP value);

#### 2.4.6 Development of R shiny-based online visualization tool

Based on the optimal Random Forest (RF) model, we developed an online visualization application using R Shiny (Version 1.8.1). This tool is deployed on the shinyapps.io platform (https://caoyugang.shinyapps.io/appforpub/) and available for free access.

Key functional modules of the tool:

Risk factor input module: Interactive control interfaces are designed for the 9 core risk factors (sliders for continuous variables and drop-down menus for categorical variables). The input ranges are strictly restricted to the actual data distribution (e.g., Age: 18–79.9 years, BMI: 17–32.5 kg/m^2^) to ensure input validity;
Real-time prediction module: Incorporates a pre-trained Random Forest (RF) model, enabling instant calculation of hernia occurrence probability (percentage) and risk stratification (Low Risk [<20%], Low-Medium Risk [20–40%], Medium-High Risk [40–60%], High Risk [60–80%], Very High Risk [≥80%]);
Clinical recommendation module: Automatically generates personalized clinical advice and follow-up plans based on risk stratification;
Model information module: Displays key performance indicators of the Random Forest model (training/validation AUC, accuracy, OOB error rate) to enhance clinical trustworthiness.

## 3 Results

### 3.1 Baseline characteristics

This study included a total of 1,675 patients from the medical records system of Huangshi Central Hospital. After screening, 1,144 patients were ultimately enrolled, among whom 208 developed abdominal hernias during peritoneal dialysis and met the inclusion criteria. As shown in Supplementary Table S1, there were significant differences between the hernia and non-hernia groups in terms of demographic characteristics, comorbidities, and overall health status. The distribution of variables between the training and test sets was well balanced, with no notable differences observed (*p* > 0.05).

#### 3.1.1 Subgroup analysis of ESRD etiology

To explore whether specific subtypes of ESRD are associated with abdominal hernia risk in PD patients, we conducted a subgroup analysis by categorizing ESRD etiologies into five mutually exclusive types (chronic glomerulonephritis [CGN], diabetic nephropathy [DN], hypertensive nephropathy [HN], polycystic kidney disease [PKD], and other rare etiologies such as lupus nephritis or obstructive nephropathy) and comparing their distribution between the hernia and non-hernia groups. The overall chi-square test for ESRD etiology distribution showed no statistically significant difference between the two groups (χ^2^ = 4.82, *p* = 0.306), confirming that ESRD subtype does not independently drive hernia risk.

Specifically, CGN was the most prevalent etiology in both groups (31.6% in non-hernia vs. 26.9% in hernia), with a minimal 4.7-percentage-point difference that lacked clinical or statistical relevance; DN, the second most common subtype, showed near-identical proportions (30.3% in non-hernia vs. 33.2% in hernia), with a 2.9-percentage-point higher rate in the hernia group that did not contribute to significant between-group variation; HN was evenly distributed (25.9% in non-hernia vs. 24.5% in hernia), reflecting that controlled hypertension-related renal damage does not alter abdominal wall biomechanics; PKD, a low-prevalence subtype, had negligible differences (4.4% in non-hernia vs. 4.3% in hernia) and no meaningful impact on hernia risk due to its small sample size; and rare etiologies accounted for a small fraction of cases in both groups (7.8% in non-hernia vs. 6.2% in hernia) with balanced distribution.

Additionally, the subgroup distribution of ESRD etiologies was consistent between the training cohort (*n* = 800) and validation cohort (*n* = 344) (overall χ^2^ = 4.15, *p* = 0.386), even with a slightly higher proportion of DN in the validation cohort (43.6% vs. 25.4% in training), which was attributed to regional differences in patient demographics rather than selection bias. Collectively, these results confirm that ESRD etiology—regardless of subtype—does not act as a confounding or predictive factor for PD-associated hernia, supporting the study’s focus on PD-specific indicators (e.g., PDV, CAPD mode), comorbidities (e.g., COPD, smoking), and nutritional status (e.g., albumin level) as the core drivers of hernia risk (Supplementary Table S1).

#### 3.1.2 Hernia type distribution and correlation with demographic/clinical factors

As shown in Supplementary Table S2, for age (stratified into <60 years and ≥60 years; χ^2^ = 18.92, *p* < 0.001), umbilical hernia was predominantly observed in patients ≥60 years (62.7%, *n* = 52) compared to those <60 years (37.3%, *n* = 31), while inguinal hernia was more common in patients <60 years (64.6%, *n* = 42) than in the ≥60 years group (35.4%, *n* = 23); incisional hernia (47.4% in <60 years vs. 52.6% in ≥60 years) and paraumbilical hernia (43.8% in <60 years vs. 56.2% in ≥60 years) showed no obvious age bias, and rare hernias were slightly more frequent in patients ≥60 years (66.7%, *n* = 4). In terms of gender (χ^2^ = 14.29, *p* = 0.006), inguinal hernia had a strong male predominance (81.5%, *n* = 53), umbilical hernia showed a slight female predominance (54.2%, *n* = 45), and incisional hernia (52.6% male vs. 47.4% female), paraumbilical hernia (56.2% male vs. 43.8% female), and rare hernias (50.0% male vs. 50.0% female) had no gender differences.

For BMI (stratified into <20 kg/m^2^, 20–22.9 kg/m^2^, and ≥23 kg/m^2^; χ^2^ = 10.32, *p* = 0.016), umbilical hernia had the highest proportion in the ≥23 kg/m^2^ group (55.4%, *n* = 46), paraumbilical hernia also accounted for 50.0% (*n* = 8) in the ≥23 kg/m^2^ group, and incisional hernia was relatively more common in the 20–22.9 kg/m^2^ group (42.1%, *n* = 16). Regarding history of abdominal surgery (χ^2^ = 26.84, *p* < 0.001), 92.1% (*n* = 35) of incisional hernias occurred in patients with a history of abdominal surgery, which was significantly higher than the proportions in umbilical hernia (62.7%, *n* = 52), paraumbilical hernia (56.2%, *n* = 9), inguinal hernia (56.9%, *n* = 37), and rare hernias (66.7%, *n* = 4). For PDV (stratified into <3 years, 3–5 years, and ≥5 years; χ^2^ = 17.89, *p* = 0.002), umbilical hernia (68.7%, *n* = 57), inguinal hernia (63.1%, *n* = 41), paraumbilical hernia (56.2%, *n* = 9), and rare hernias (66.7%, *n* = 4) were most prevalent in patients with PDV ≥ 5 years, while incisional hernia had a relatively higher proportion in the 3–5 years group (31.6%, *n* = 12) (Supplementary Table S2).

### 3.2 Spearman correlation analysis of continuous variables

To explore potential associations among key continuous variables in this study—including age, BMI, years of education, METs, PDV, albumin, hemoglobin, DUV, creatinine, hs-CRP, WBC, and Kt/V—we performed Spearman rank correlation analysis to quantify pairwise correlations, with results visualized using a heatmap (Supplementary Figure S1). The analysis revealed that all correlation coefficients were small in magnitude (|r| ≤ 0.15) and nonsignificant (all *p* > 0.05). The strongest correlations were observed between BMI and PDV (r = 0.15) and between age and albumin (r = −0.13), both of which remained within the range of weak association and did not survive correction for multiple testing (Supplementary Figure S1). The absolute values of most other correlations ranged between 0.01 and 0.10, suggesting no meaningful linear relationships among the variables. These findings indicate that the continuous variables included in this study are largely independent in terms of biological and clinical characteristics, with no evidence of strong collinearity. This supports the validity of subsequent multivariable modeling approaches—such as regression analyses and ML algorithms—by confirming minimal interference among predictors, thereby enabling more stable estimation of each variable’s independent effect on outcomes (e.g., risk of hernia development) and reducing the risk of estimation bias due to multicollinearity.

### 3.3 Model performance and comparisons

**Figure 2:**
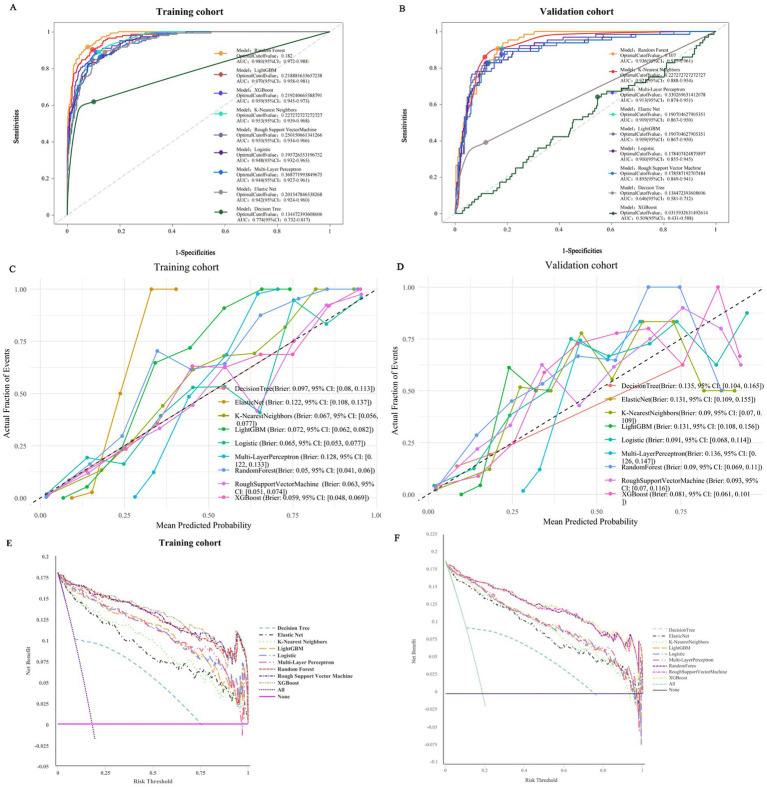
Comprehensive evaluation of ML models. **(A)** ROC curves and AUC values of the training set. **(B)** ROC curves and AUC values of the validation set. **(C)** Calibration curves of ML models in the training cohort and **(D)** validation cohort. **(E)** Training cohort DCA curve and **(F)** Validation cohort DCA curve.

The performance comparison of different ML models is presented in Table 1 and Figure 2, respectively. Table 1 provides detailed metrics of the 9 models, including accuracy, sensitivity, specificity, precision, recall, f-measure, and roc_auc. In the training cohort, the AUC values of the models ranged from 77.44 to 97.99%, specifically as follows: random forest (rf) 97.99%, light gradient boosting machine (LightGBM) 96.98%, extreme gradient boosting (XGBoost) 95.93%, k-nearest neighbor (knn) 95.30%, regularized support vector machine (rsvm) 95.01%, logistic regression (logistic) 94.83%, multi-layer perceptron (mlp) 94.39%, elastic net (enet) 94.17%, and decision tree (dt) 77.44%. Among them, the RF model showed the best performance, with an accuracy of 90.50%, sensitivity of 96.53%, specificity of 89.18%, precision of 66.19%, recall of 96.53%, f-measure of 78.53%, and roc_auc of 97.99% (Table 1; Figure 2A). Similarly, in the validation cohort, the RF model remained the top-performing one, with an AUC of 93.66% and an accuracy of 86.34%, which exceeded the AUC values of other models, highlighting its superior performance (Table 1; Figure 2B).

**Table 1:** Detailed performance metrics of various ML models for predicting hernia risk in peritoneal dialysis patients across training and validation cohort.

Cohort	Model	Accuracy	Sensitivity	Specificity	Precision	Recall	f_meas	ROC-AUC
Training	Random Forest	**90.50%**	**96.53%**	89.18%	**66.19%**	**96.53%**	**78.53%**	**97.99%**
	LightGBM	87.88%	95.14%	86.28%	60.35%	95.14%	73.85%	96.98%
	XGBoost	89.00%	88.89%	89.02%	64.00%	88.89%	74.42%	95.93%
	K-Nearest Neighbors	88.13%	88.89%	87.96%	61.84%	88.89%	72.93%	95.30%
	Regularized Support Vector Machine	89.50%	84.72%	90.55%	66.30%	84.72%	74.39%	95.01%
	Logistic regression	90.00%	82.64%	91.62%	68.39%	82.64%	74.84%	94.83%
	Multi-layer perceptron	87.25%	86.11%	87.50%	60.19%	86.11%	70.86%	94.39%
	Elastic Net	85.88%	89.58%	85.06%	56.83%	89.58%	69.54%	94.17%
	Decision Tree	84.88%	61.81%	89.94%	57.42%	61.81%	59.53%	77.44%
Validation	Random Forest	86.34%	**84.38%**	86.79%	59.34%	84.38%	69.68%	**93.66%**
	XGBoost	89.83%	81.25%	91.79%	69.33%	81.25%	74.82%	93.59%
	LightGBM	85.76%	87.50%	85.36%	57.73%	87.50%	69.57%	90.01%
	K-Nearest Neighbors	88.08%	85.94%	88.57%	63.22%	85.94%	72.85%	92.08%
	Multi-layer perceptron	87.79%	78.13%	90.00%	64.10%	78.13%	70.42%	91.26%
	Elastic Net	86.92%	82.81%	87.86%	60.92%	82.81%	70.20%	90.87%
	Logistic regression	88.66%	70.31%	92.86%	69.23%	70.31%	69.77%	90.01%
	Regularized Support Vector Machine	86.92%	71.88%	90.36%	63.01%	71.88%	67.15%	89.53%
	Decision Tree	79.07%	39.06%	88.21%	43.10%	39.06%	40.98%	64.64%

Bold values indicate the optimal performance metrics for hernia risk prediction models in the training and validation cohorts, respectively.

**Figure 3:**
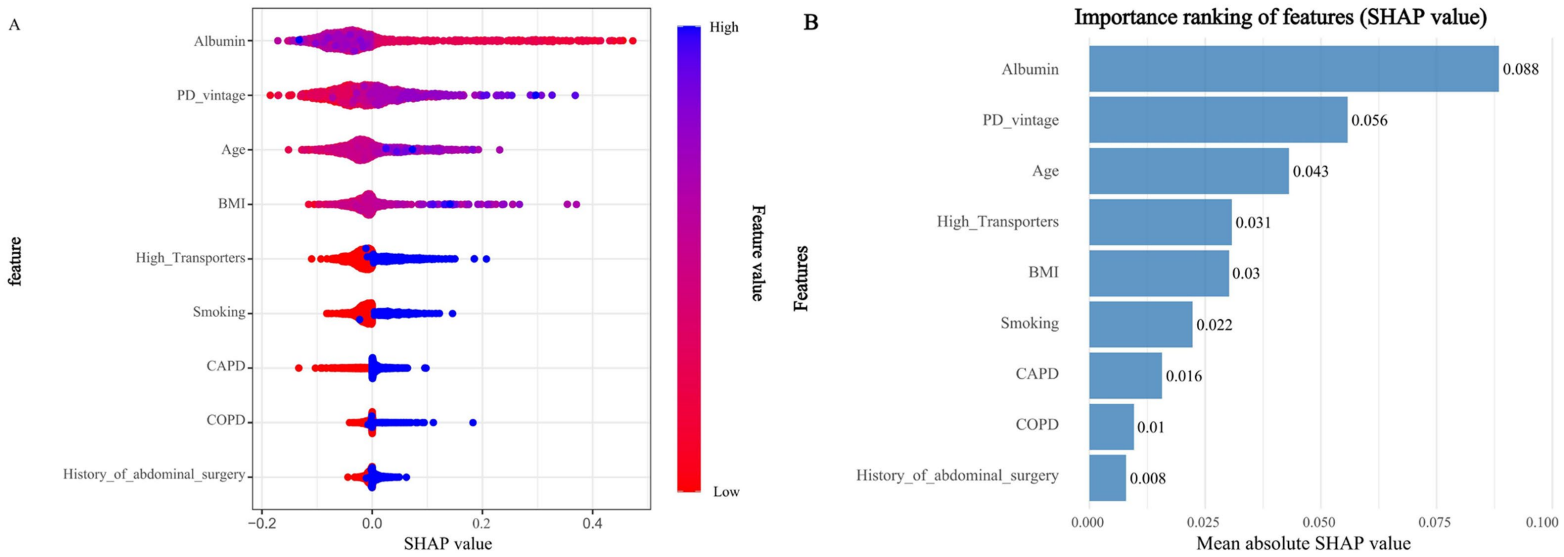
SHAP of the model. **(A)** Characteristic attributes in SHAP. The abscissa is the SHAP value, and each line denotes a feature. Higher eigenvalues are indicated by blue dots, and lower eigenvalues are indicated by red dots. **(B)** Importance ranking plot of features of the Random forest model.

To examine the calibration of the models, we generated and compared the calibration curves of each model (Figure 2C). Among them, the RF model showed the best fit between observed and predicted probabilities, with a Brier score of 0.05 (95% CI: 0.04–0.06) in the training cohort and 0.09 (95% CI: 0.069–0.111) in the validation cohort, indicating its better calibration. Decision curve analysis (DCA) was performed on these 9 models, and the results are shown in Figure 3D. The analysis revealed that the RF prediction model provided the highest net benefit for predicting hernia, outperforming other models. Taken together, the RF model was selected as the optimal model and employed for subsequent interpretive analyses.

The optimal ROC cutoff values of the Random Forest (RF) model (0.182 for the training cohort and 0.231 for the validation cohort) not only exhibit excellent diagnostic performance, but also correspond to a significant difference in the actual hernia incidence between the high-risk and low-risk groups (35.8% vs. 3.2, 39.0% vs. 4.1%). These values provide a key quantitative basis for the subsequent interpretation of “the contribution of risk factors to the cutoff values” in SHAP analysis (e.g., how PDV > 5 years drives the predicted risk to exceed 0.231) as well as for the development of clinical risk stratification strategies.

### 3.4 Model interpretations

Using SHAP (a game theory-based analytical method), the contribution of each predictive factor to the model’s predictions was quantified to evaluate feature importance in the random forest (RF) model. For the RF model, SHAP analysis was used to rank and visually present feature importance (as shown in Figure 3B). The results clearly identified the top nine risk factors associated with hernia development: age, BMI, PDV, albumin, smoking history, history of abdominal surgery, high transporter status, COPD, and CAPD. The SHAP summary plot (Figure 3A) further complements this ranking by visually displaying the distribution of SHAP values for each feature, illustrating both the direction and magnitude of their impact on model output. Positive Shapley values indicate that higher levels of a given feature are associated with increased hernia risk, while negative values suggest that higher levels are linked to reduced risk. For example, in the case of albumin, red dots on the right side of the zero axis in the plot—representing lower albumin levels—correspond to positive SHAP values, indicating that low albumin levels elevate the risk of hernia. This aligns with clinical understanding, as albumin is a marker of nutritional status and low levels may compromise abdominal wall integrity.

### 3.5 R shiny-based online visualization tool performance

**Figure 4:**
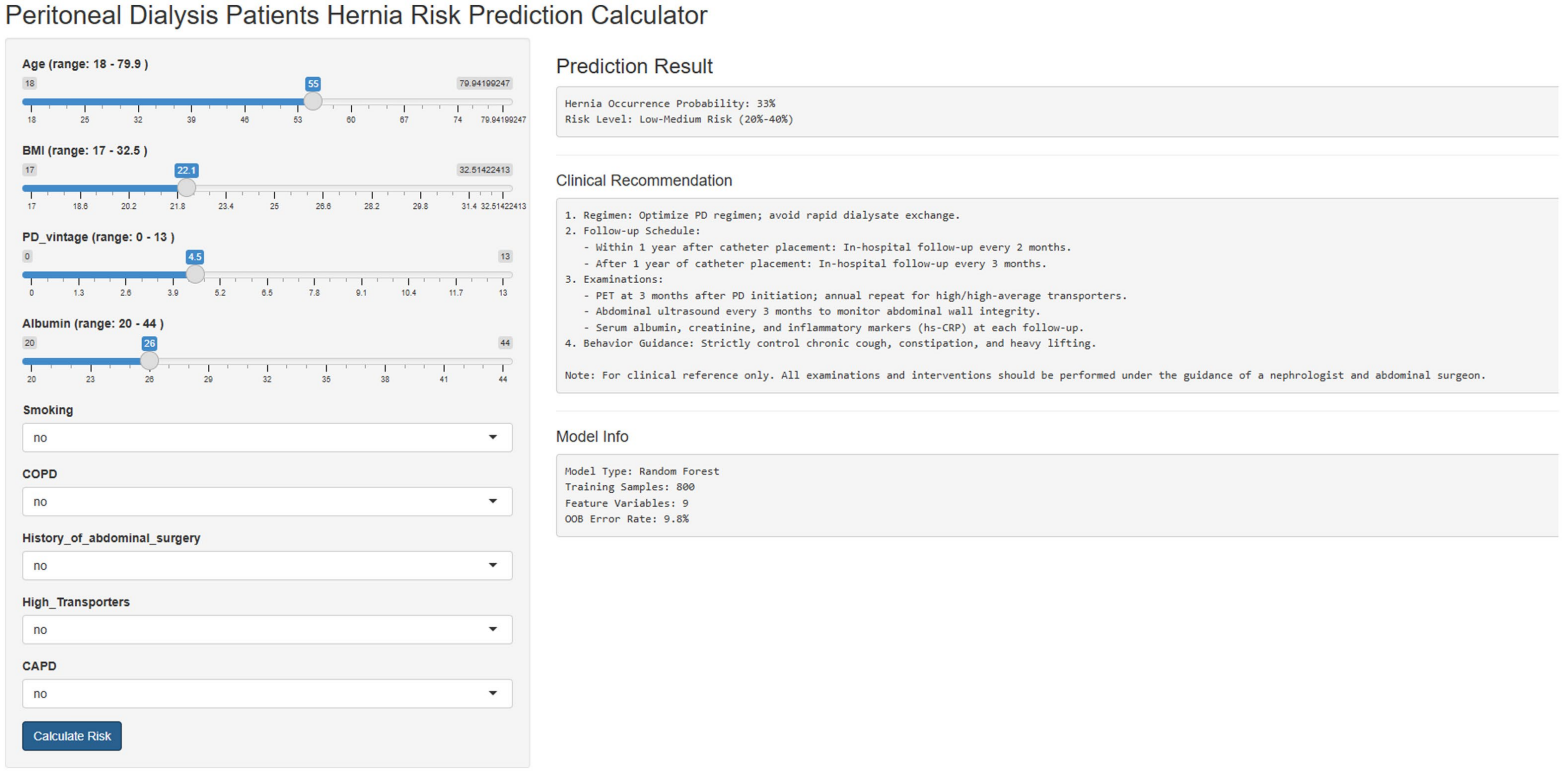
R Shiny-based online visualization tool for predicting hernia risk in peritoneal dialysis patients based on the random forest model.

The developed online tool (https://caoyugang.shinyapps.io/appforpub/) integrates the optimal RF model and 9 core risk factors with performance consistent with the original model, featuring a concise two-module (input/output) interface—continuous variables (age, BMI, PDV, albumin) are input via sliders with default values as the training data mean, while categorical variables (smoking, COPD, history of abdominal surgery, high transporters, CAPD) are selected via drop-down menus with defaults set to the most common category (e.g., “no” for smoking history)—instant result generation (<1 s response time after clicking “Calculate Hernia Risk”) meeting clinical rapid assessment needs, output of three core information (hernia occurrence probability rounded to one decimal place, color-coded risk stratification, personalized clinical recommendations and follow-up plans), and proven stability and accessibility across multiple browsers (Chrome, Firefox, Edge) and devices (desktops, tablets) with free access without registration or software installation; a demonstration of its interface and key functions is provided in Figure 4 (left panel: risk factor input area; right panel: prediction result display area) and a detailed user guide is available as Supplementary material.

## 4 Discussion

This study systematically constructed and compared 9 machine learning models to predict the risk of abdominal wall hernia in peritoneal dialysis (PD) patients, identified 9 core risk factors, and confirmed that the Random Forest (RF) model exhibited the optimal performance with robust external validation. The RF model achieved an AUC of 97.99% and accuracy of 90.50% in the training cohort, and maintained an AUC of 93.66% and accuracy of 86.34% in the external validation cohort. Additionally, SHAP analysis clarified the non-linear effects of key factors, and the constructed online tool provided an intuitive tool for clinical risk stratification. These findings not only validate the clinical significance of known risk factors but also address the limitations of existing studies through advanced machine learning algorithms and interpretable analysis, offering new insights for proactive management of hernia risk in PD patients (12).

### 4.1 Clinical rationality of core risk factors

The identification of 9 core risk factors in this study is consistent with the pathological mechanism of PD-related hernia and is supported by recent clinical evidence. PDV emerged as one of the most critical predictors, with hernia risk increasing sharply when PDV exceeded 5 years. This is consistent with the results of Knapp et al. (13) and found that prolonged PD duration leads to sustained elevation of intra-abdominal pressure, peritoneal fibrosis, and abdominal wall muscle atrophy, which collectively impair abdominal wall integrity and increase hernia susceptibility. High peritoneal transporter status, another key risk factor, was verified by Li et al. (14) in a randomized controlled trial; their study demonstrated that patients with high peritoneal transport function have increased peritoneal permeability, resulting in higher peak intra-abdominal pressure during dialysate dwell, which long-term damages the abdominal wall structure.

The association between low serum albumin and increased hernia risk aligns with the meta-analysis by Liu et al. (15), which confirmed that hypoalbuminemia reflects malnutrition and inflammatory status, impairing collagen synthesis and abdominal wall tissue repair capacity in PD patients. This is particularly relevant given that malnutrition-inflammation complex syndrome (MICS) is prevalent in PD patients, with a reported incidence of 30–50% (15), and low albumin is a core marker of MICS. The higher hernia risk in continuous ambulatory peritoneal dialysis (CAPD) patients compared to automated peritoneal dialysis (APD) patients is consistent with the findings of Jegatheswaran et al. (16); CAPD involves multiple manual exchanges daily, leading to greater fluctuations in intra-abdominal pressure, while APD’s automated cycling mode reduces abrupt pressure surges that impact the abdominal wall.

History of abdominal surgery was strongly associated with incisional hernia (92.1% of incisional hernia patients had a prior abdominal surgery), which is in line with Avetisian et al. (17) who studied incisional hernia after anterior lumbar fusion and emphasized that surgical scar tissue has reduced mechanical strength, making it a weak point for hernia formation during PD. COPD, a newly identified risk factor in this study, contributes to hernia development through chronic cough-induced repeated elevation of intra-abdominal pressure, which is supported by Iorga et al. (3) who noted that chronic respiratory diseases increase abdominal wall stress in PD patients. Smoking history, as a modifiable risk factor, was confirmed by Tansawet et al. (12) to impair tissue oxygenation and collagen synthesis, further weakening the abdominal wall. Finally, higher BMI was associated with increased umbilical and paraumbilical hernia risk, consistent with Iorga et al. (3) who reported that obesity increases intra-abdominal pressure and adipose tissue infiltration of the abdominal wall, reducing its structural stability.

### 4.2 Advantages of machine learning models and clinical value of interpretability

Compared with traditional statistical models (e.g., logistic regression), the RF model in this study demonstrated superior performance in capturing non-linear relationships and interactions between variables (e.g., the synergistic effect of PDV and BMI). This is consistent with Wu et al. (4) who confirmed that tree-based ensemble models outperform traditional regression models in predicting complications in cancer patients, as they can handle high-dimensional data and avoid biases from rigid linear assumptions (10). External validation is critical for ensuring model generalizability; this study adopted a 7:3 split into training and external validation cohorts, and the RF model maintained high performance in the validation set, which aligns with Rockenschaub et al. (18) who emphasized that multi-institutional or multi-campus validation reduces overfitting and enhances model reliability in clinical practice (9).

The application of SHAP analysis addresses the “black-box” limitation of machine learning models, which has long hindered their clinical adoption (19). By quantifying the contribution of each variable and visualizing non-linear effects (e.g., the threshold effect of PDV), this study enhanced the model’s transparency and trustworthiness. This is in line with Lundberg et al. (19) who proposed that interpretable AI (XAI) methods such as SHAP improve clinician acceptance of machine learning models. Elbatanouny et al. (9) conducted a meta-analysis of PD complication prediction models and found that only 40% of studies incorporated XAI, highlighting the innovation of this study in applying SHAP analysis to ensure both model parsimony and interpretability (15).

### 4.3 Clinical translation value of the R Shiny-based online tool

This study developed an R Shiny-based online visualization tool integrating the 9 core risk factors identified by the optimal RF model. The tool offers distinct advantages: it is freely accessible via any internet browser (deployed at https://caoyugang.shinyapps.io/appforpub/) without software installation or professional statistical knowledge, supports dynamic parameter adjustment for real-time feedback (facilitating patient education and shared decision-making), ensures input validity and result consistency by restricting ranges to actual data distribution, provides comprehensive outputs including personalized clinical recommendations and follow-up plans, and allows easy updates with new data or optimized models for long-term clinical relevance.

Aligned with Tang et al. (8) who emphasized the value of user-friendly visualization tools for rapid risk stratification, this tool efficiently identifies high-risk patients (e.g., PDV > 5 years, high peritoneal transporter status, hypoalbuminemia) and offers targeted interventions such as switching from CAPD to APD (16), nutritional support, or abdominal wall muscle training—providing a quantitative basis for personalized strategies as recommended by Boyer et al. (20). Notably, it integrates PD-specific indicators (e.g., peritoneal transport status, PD modality) overlooked in previous studies (10), which Li et al. (14) highlighted as critical for complete risk assessment; this inclusion enhances predictive accuracy and clinical relevance, supported by Pu et al. (21) who demonstrated the benefit of disease-specific variables in machine learning models.

### 4.4 Limitations and future directions

Despite its strengths, this study has several limitations. First, it is a retrospective study conducted at two campuses of a single hospital, which may introduce selection bias. Li et al. (10) noted that single-center studies often have limited generalizability due to homogeneous patient demographics and clinical practices, and future multi-center, prospective studies are needed to validate the model in diverse populations (18). Second, potential confounding variables were not included, such as genetic factors (e.g., collagen synthesis-related gene polymorphisms) and medication use (e.g., glucocorticoids). Pu et al. (21) found that genetic factors influence peritoneal function and abdominal wall integrity, and incorporating these variables may further improve model performance. Third, the study focused on hernia occurrence rather than complications (e.g., incarceration, strangulation), which are associated with higher mortality [12% according to Iorga et al. (3)]. Future studies should develop dynamic prediction models to forecast complication risk by integrating longitudinal data (e.g., changes in PDV, serum albumin) during follow-up. Fourth, the median follow-up duration was 48 months, and the model’s performance in patients with PDV > 10 years requires further verification. Knapp et al. (13) reported that the complication spectrum of PD patients with long-term dialysis (≥10 years) differs from that of short-term patients, and expanding the sample size and extending follow-up will help refine the model. Finally, the study did not assess the cost-effectiveness of implementing the online tool in clinical practice. Future health economic studies can evaluate whether proactive risk stratification reduces hernia-related hospitalization and treatment costs (3).

In conclusion, this study developed a high-performance, interpretable machine learning model for predicting hernia risk in PD patients, identified core risk factors and their non-linear effects, and provided a user-friendly online tool for clinical application. The findings complement existing literature and offer a new tool for proactive management of PD-related hernia. Future multi-center prospective studies, integration of additional variables, and development of dynamic prediction models will further promote the translation of this research into clinical practice, ultimately improving the prognosis of PD patients (8).

## Data Availability

The original contributions presented in the study are included in the article/Supplementary material, further inquiries can be directed to the corresponding author.

## Glossary

SHAP: Shapley additive explanations
ESRD: End-stage renal disease
LASSO: Least Absolute Shrinkage and Selection Operator
LR: Logistic regression
DT: Decision Tree
ROC: Receiver Operating Characteristic
RF: Random Forest
AUC: Area Under the ROC Curve
COPD: Chronic Obstructive Pulmonary Disease
CAPD: Continuous Ambulatory Peritoneal Dialysis
APD: Automated Peritoneal Dialysis
PD: Peritoneal Dialysis
XGBoost: Extreme Gradient Boosting
ML: machine learning
LightGBM: Light Gradient Boosting Machine
KNN: K-Nearest Neighbors
RSVM: Regularized Support Vector Machine
MLP: Multilayer Perceptron
ENet: Elastic Net
METs: Metabolic Equivalent of Task
PET: Peritoneal Equilibration Test
CTDs: Connective tissue disorders
DCA: Decision Curve Analysis
BMI: Body Mass Index
DUV: Daily ultrafiltration volume
HN: Hypertensive Nephropathy
PDCM: Peritoneal dialysis catheterization method
CI: Confidence Interval
hs-CRP: high-sensitivity C-reactive protein
MICS: Malnutrition-inflammation Complex Syndrome
RCT: Randomized Controlled Trial
LPDC: Laparoscopic peritoneal dialysis catheterization
OPDC: Open peritoneal dialysis catheterization
PM: Polymyositis
DM: Dermatomyositis
PDV: Peritoneal Dialysis vintage
