# Supplementary material for: Machine learning-based prediction of hernia risk in peritoneal dialysis patients: a comparative study of models and SHAP-driven interpretability analysis
Source: Front Med (Lausanne). 2026 Mar 4;13:1687055. doi: 10.3389/fmed.2026.1687055 (PMC12995620; doi:10.3389/fmed.2026.1687055)
Supplement: Supplementary file 1 [file Table_1.docx]

Supplementary Material

Table S1. Comparison of characteristics between non-hernia and hernia groups, and between training and validation cohorts

| **Characteristic** | **Non-Hernia (N=936)** | **Hernia (N=208)** | **Statistic** | **P** | **Training cohorts (N=800)** | **Validation cohorts (N=344)** | **Statistic** | **P** |
| --- | --- | --- | --- | --- | --- | --- | --- | --- |
| **Age** | 52.9 (14.6) | 62.6 (11.6) | W=55419 | <0.001 | 54.6(13.5) | 56.3(15.5) | W=135252 | 0.647 |
| **BMI** | 21.7 (2.69) | 23.5 (3.21) | W=66006 | <0.001 | 22.1(2.80) | 22.8(3.03) | W=142372 | 0.352 |
| **PDV** | 3.91 (2.83) | 6.85 (2.60) | W=45374 | <0.001 | 4.52(3.02) | 4.28(2.99) | W=143902 | 0.216 |
| **Creatinine** | 804 (230) | 814 (234) | W=94987 | 0.585 | 804(232) | 809(229) | W=137314 | 0.955 |
| **Albumin** | 37.9 (5.95) | 31.7 (5.87) | W=154135 | <0.001 | 36.5(6.26) | 37.3(6.69) | W=130345 | 0.157 |
| **Hemoglobin** | 9.90 (2.39) | 9.93 (2.57) | W=96707 | 0.883 | 10.0(2.37) | 9.68(2.53) | W=147398 | 0.0559 |
| **DUV** | 1220 (385) | 1170 (361) | W=103650 | 0.143 | 1230(374) | 1190(395) | W=146112 | 0.0967 |
| **Years of Education** | 7.21 (4.59) | 7.17 (4.81) | W=97601 | 0.952 | 7.31(4.59) | 6.97(4.71) | W=143338 | 0.262 |
| **Kt/V** | 4.81 (1.87) | 5.02 (1.79) | W=90880 | 0.134 | 4.88(1.88) | 4.78(1.79) | W=141573 | 0.438 |
| **WBC** | 6.48 (1.73) | 6.73 (1.69) | W=89250 | 0.0604 | 6.52(1.72) | 6.54(1.73) | W=136852 | 0.884 |
| **hs-CRP** | 7.62 (4.46) | 7.99 (4.50) | W=92652 | 0.276 | 7.81(4.46) | 7.41(4.48) | W=144774 | 0.162 |
| **Diabetes** |  |  |  |  |  |  |  |  |
| No | 644(68.8%) | 134(64.4%) | χ²=1.31 | 0.253 | 554(69.3%) | 224(65.1%) | χ²=1.7 | 0.192 |
| Yes | 292(31.2%) | 74(35.6%) |  |  | 246(30.8%) | 120(34.9%) |  |  |
| **Smoking** |  |  |  |  |  |  |  |  |
| No | 725(77.5%) | 116(55.8%) | χ²=40 | <0.001 | 581(72.6%) | 260(75.6%) | χ²=0.933 | 0.334 |
| Yes | 211(22.5%) | 92(44.2%) |  |  | 219(27.4%) | 84(24.4%) |  |  |
| **Gender** |  |  |  |  |  |  |  |  |
| female | 397(42.4%) | 108(51.9%) | χ²=5.86 | 0.0155 | 349(43.6%) | 156(45.3%) | χ²=0.224 | 0.636 |
| male | 539(57.6%) | 100(48.1%) |  |  | 451(56.4%) | 188(54.7%) |  |  |
| **Residence** |  |  |  |  |  |  |  |  |
| City | 637(68.1%) | 142(68.3%) | χ²=<0.001 | 1 | 544(68.0%) | 235(68.3%) | χ²=0.00125 | 0.972 |
| Rural areas | 299(31.9%) | 66(31.7%) |  |  | 256(32.0%) | 109(31.7%) |  |  |
| **History of abdominal surgery** |  |  |  |  |  |  |  |  |
| No | 611(65.3%) | 110(52.9%) | χ²=10.7 | 0.00108 | 506(63.3%) | 215(62.5%) | χ²=0.0303 | 0.862 |
| Yes | 325(34.7%) | 98(47.1%) |  |  | 294(36.8%) | 129(37.5%) |  |  |
| **Marital status** |  |  |  |  |  |  |  |  |
| No | 177(18.9%) | 36(17.3%) | χ²=0.192 | 0.661 | 147(18.4%) | 66(19.2%) | χ²=0.0578 | 0.81 |
| Yes | 759(81.1%) | 172(82.7%) |  |  | 653(81.6%) | 278(80.8%) |  |  |
| **Low Transporters** |  |  |  |  |  |  |  |  |
| No | 699(74.7%) | 192(92.3%) | χ²=29.7 | <0.001 | 614(76.8%) | 277(80.5%) | χ²=1.78 | 0.183 |
| Yes | 237(25.3%) | 16(7.7%) |  |  | 186(23.3%) | 67(19.5%) |  |  |
| **Low Average Transporters** |  |  |  |  |  |  |  |  |
| No | 719(76.8%) | 170(81.7%) | χ²=2.1 | 0.148 | 615(76.9%) | 274(79.7%) | χ²=0.916 | 0.339 |
| Yes | 217(23.2%) | 38(18.3%) |  |  | 185(23.1%) | 70(20.3%) |  |  |
| **High Transporters** |  |  |  |  |  |  |  |  |
| No | 713(76.2%) | 78(37.5%) | χ²=118 | <0.001 | 563(70.4%) | 228(66.3%) | χ²=1.7 | 0.192 |
| Yes | 223(23.8%) | 130(62.5%) |  |  | 237(29.6%) | 116(33.7%) |  |  |
| **High Average Transporters** |  |  |  |  |  |  |  |  |
| No | 677(72.3%) | 184(88.5%) | χ²=22.9 | <0.001 | 608(76.0%) | 253(73.5%) | χ²=0.652 | 0.42 |
| Yes | 259(27.7%) | 24(11.5%) |  |  | 192(24.0%) | 91(26.5%) |  |  |
| **Hypertension** |  |  |  |  |  |  |  |  |
| No | 416(44.4%) | 92(44.2%) | χ²=<0.001 | 1 | 360(45.0%) | 148(43.0%) | χ²=0.305 | 0.953 |
| Yes | 520(55.6%) | 116(55.8%) |  |  | 440(55.0%) | 196(57.0%) |  |  |
| **Heart disease** |  |  |  |  |  |  |  |  |
| No | 747(79.8%) | 160(76.9%) | χ²=0.695 | 0.404 | 633(79.1%) | 274(79.7%) | χ²=0.0148 | 0.903 |
| Yes | 189(20.2%) | 48(23.1%) |  |  |  |  |  |  |
| **CAPD** |  |  |  |  |  |  |  |  |
| No | 272(29.1%) | 7(3.4%) | χ²=59.5 | <0.001 | 199(24.9%) | 80(23.3%) | χ²=0.26 | 0.61 |
| Yes | 664(70.9%) | 201(96.6%) |  |  | 601(75.1%) | 264(76.7%) |  |  |
| **APD** |  |  |  |  |  |  |  |  |
| No | 664(70.9%) | 199(95.7%) | χ²=54.9 | <0.001 | 600(75.0%) | 263(76.5%) | χ²=0.201 | 0.654 |
| Yes | 272(29.1%) | 9(4.3%) |  |  | 200(25.0%) | 81(23.5%) |  |  |
| **PDCM** |  |  |  |  |  |  |  |  |
| Open surgery | 648(69.2%) | 107(51.4%) | χ²=23.2 | <0.001 | 527(65.9%) | 228(66.3%) | χ²=0.00413 | 0.949 |
| Laparoscopic surgery | 288(30.8%) | 101(48.6%) |  |  | 273(34.1%) | 116(33.7%) |  |  |
| **METs** |  |  |  |  |  |  |  |  |
| Light Labor | 281(30.0%) | 65(31.3%) | χ²=2.47 | 0.481 | 234(29.3%) | 112(32.6%) | χ²=3.59 | 0.309 |
| Moderate Labor | 257(27.5%) | 47(22.5%) |  |  | 211(26.4%) | 93(27.0%) |  |  |
| Heavy Labor | 212(22.6%) | 48(23.1%) |  |  | 180(22.5%) | 80(23.3%) |  |  |
| Very Heavy Labor | 186(19.9%) | 48(23.1%) |  |  | 175(21.9%) | 59(17.2%) |  |  |
| **COPD** |  |  |  |  |  |  |  |  |
| No | 838(89.5%) | 145(69.7%) | χ²=53.6 | <0.001 | 694(86.8%) | 289(84.0%) | χ²=1.27 | 0.259 |
| Yes | 98(10.5%) | 63(30.3%) |  |  | 106(13.3%) | 55(16.0%) |  |  |
| **Connective tissue disease** |  |  |  |  |  |  |  |  |
| No | 861(92.0%) | 163(78.4%) | χ²=32.2 | <0.001 | 716(89.5%) | 308(89.5%) | χ²=<0.001 | 1 |
| Yes | 75(8.0%) | 45(21.6%) |  |  | 84(10.5%) | 36(10.5%) |  |  |
| **Subgroup Analysis of ESRD Etiology** |  |  |  |  |  |  |  |  |
| Diabetic Nephropathy (DN) | 284(30.3%) | 69 (33.2%) | χ²=4.82 | 0.306 | 203(25.4%) | 150(43.6%) | χ²=4.15 | 0.386 |
| Hypertensive Nephropathy (HN) | 242(25.9%) | 51 (24.5%) |  |  | 195(24.4%) | 98 (28.5%) |  |  |
| Chronic Glomerulonephritis (CGN) | 296(31.6%) | 56 (26.9%) |  |  | 240(30.0%) | 112(32.6%) |  |  |
| Polycystic Kidney Disease (PKD) | 41 (4.4%) | 9 (4.3%) |  |  | 34 (4.3%) | 16 (4.7%) |  |  |
| Other Etiologies (e.g., lupus nephritis, obstructive nephropathy) | 73 (7.8%) | 13 (6.2%) |  |  | 128(16.0%) | 20 (5.8%) |  |  |

Note: 1. This table presents demographic, clinical, and laboratory indicators related to hernia risk, including both core candidate variables and subgroup stratification of partial variables (e.g., "Subgroup Analysis of ESRD Etiology" is a stratified description of the "ESRD etiology" variable, not an independent variable). The 29 core candidate variables referenced in Section 3.3 include the following categories: (1) Continuous variables (8 items): Age, BMI, PDV, serum albumin, hemoglobin, DUV, creatinine, Kt/V; (2) Categorical variables (21 items): Smoking history, diabetes, history of abdominal surgery, peritoneal transport status (low/ low-average/ high-average/ high), PD modality (CAPD/APD), PD catheterization method (open/laparoscopic), COPD, connective tissue disease, gender, residence, marital status, years of education, METs level, hypertension, heart disease, WBC, hs_CRP, etc. 2. Data are presented as n (%) or mean (standard deviation).
