# Supplementary material for: Machine learning-based prediction of hernia risk in peritoneal dialysis patients: a comparative study of models and SHAP-driven interpretability analysis
Source: Front Med (Lausanne). 2026 Mar 4;13:1687055. doi: 10.3389/fmed.2026.1687055 (PMC12995620; doi:10.3389/fmed.2026.1687055)
Supplement: Supplementary file 2 [file Table_2.docx]

Supplementary Material

Table S2. Distribution of Demographic and Clinical Factors Across Different Types of Abdominal Wall Hernias in Peritoneal Dialysis Patients

| Factor | Umbilical Hernia (n=83, 40.0%) | Inguinal Hernia (n=65, 31.3%) | Incisional Hernia (n=38, 18.3%) | Paraumbilical Hernia (n=16, 7.7%) | Rare Hernia (n=6, 2.9%) | Statistic | P-value |
| --- | --- | --- | --- | --- | --- | --- | --- |
| Age |  |  |  |  |  | χ²=18.92 | <0.001 |
| <60 years | 31 (37.3%) | 42 (64.6%) | 18 (47.4%) | 7 (43.8%) | 2 (33.3%) |  |  |
| ≥60 years | 52 (62.7%) | 23 (35.4%) | 20 (52.6%) | 9 (56.2%) | 4 (66.7%) |  |  |
| Gender |  |  |  |  |  | χ²=14.29 | 0.006 |
| Male | 38 (45.8%) | 53 (81.5%) | 20 (52.6%) | 9 (56.2%) | 3 (50.0%) |  |  |
| Female | 45 (54.2%) | 12 (18.5%) | 18 (47.4%) | 7 (43.8%) | 3 (50.0%) |  |  |
| BMI |  |  |  |  |  | χ²=10.32 | 0.016 |
| <20 kg/m² | 12 (14.5%) | 10 (15.4%) | 8 (21.1%) | 2 (12.5%) | 1 (16.7%) |  |  |
| 20–22.9 kg/m² | 25 (30.1%) | 22 (33.8%) | 16 (42.1%) | 6 (37.5%) | 2 (33.3%) |  |  |
| ≥23 kg/m² | 46 (55.4%) | 33 (50.8%) | 14 (36.8%) | 8 (50.0%) | 3 (50.0%) |  |  |
| History of Abdominal Surgery |  |  |  |  |  | χ²=26.84 | <0.001 |
| No | 31 (37.3%) | 28 (43.1%) | 3 (7.9%) | 7 (43.8%) | 2 (33.3%) |  |  |
| Yes | 52 (62.7%) | 37 (56.9%) | 35 (92.1%) | 9 (56.2%) | 4 (66.7%) |  |  |
| PDV |  |  |  |  |  | χ²=17.89 | 0.002 |
| <3 years | 12(14.5%) | 11(16.9%) | 8(21.1%) | 3(18.8%) | 1(16.7%) |  |  |
| 3-5years | 14(16.9%) | 13(20.0%) | 12(31.6%) | 4(25.0%) | 1(16.7%) |  |  |
| ≥5years | 57(68.7%) | 41(63.1%) | 18(47.3%) | 9(56.2%) | 4(66.7%) |  |  |

Data are presented as n (%) .
