# Supplementary material for: Machine learning-based prediction of hernia risk in peritoneal dialysis patients: a comparative study of models and SHAP-driven interpretability analysis
Source: Front Med (Lausanne). 2026 Mar 4;13:1687055. doi: 10.3389/fmed.2026.1687055 (PMC12995620; doi:10.3389/fmed.2026.1687055)
Supplement: Supplementary file 3 [file Table_3.docx]

Supplementary Material

**Report on 5-Fold Cross-Validation Results of Machine Learning Models for Predicting Abdominal Wall Hernia in Peritoneal Dialysis Patients**

Table S3. 5-Fold Cross-Validation Results

| Model Name | AUC  (95% CI) | Accuracy (95% CI) | Sensitivity (95% CI) | Specificity (95% CI) | F1-score (95% CI) |
| --- | --- | --- | --- | --- | --- |
| Random Forest | 0.946 (0.946-0.946) | 0.901 (0.877-0.925) | 0.570 (0.491-0.649) | 0.974 (0.954-0.994) | 0.675 (0.598-0.752) |
| Logistic Regression | 0.938 (0.903-0.972) | 0.849 (0.799-0.899) | 0.847 (0.776-0.919) | 0.849 (0.803-0.895) | 0.671 (0.579-0.763) |
| Regularized Support Vector Machine | 0.927 (0.888-0.966) | 0.860 (0.813-0.907) | 0.833 (0.739-0.927) | 0.866 (0.827-0.905) | 0.683 (0.584-0.782) |
| LightGBM | 0.927 (0.899-0.954) | 0.875 (0.837-0.913) | 0.688 (0.593-0.782) | 0.916 (0.887-0.945) | 0.665 (0.568-0.762) |
| Elastic Net | 0.925 (0.896-0.955) | 0.900 (0.860-0.940) | 0.584 (0.441-0.727) | 0.970 (0.952-0.987) | 0.675 (0.534-0.816) |
| XGBoost | 0.919 (0.890-0.949) | 0.884 (0.837-0.930) | 0.680 (0.568-0.793) | 0.928 (0.893-0.964) | 0.679 (0.554-0.805) |
| Multi-layer Perceptron | 0.917 (0.898-0.936) | 0.869 (0.851-0.887) | 0.619 (0.493-0.744) | 0.924 (0.911-0.937) | 0.626 (0.550-0.703) |
| K-Nearest Neighbors | 0.881 (0.827-0.935) | 0.879 (0.856-0.901) | 0.549 (0.455-0.643) | 0.951 (0.943-0.960) | 0.618 (0.535-0.701) |
| Decision Tree | 0.786 (0.735-0.837) | 0.820 (0.798-0.842) | 0.702 (0.605-0.799) | 0.846 (0.821-0.871) | 0.583 (0.532-0.634) |

All metrics are presented as "mean (95% CI)".
