# Supplementary material for: Machine learning-based prediction of hernia risk in peritoneal dialysis patients: a comparative study of models and SHAP-driven interpretability analysis
Source: Front Med (Lausanne). 2026 Mar 4;13:1687055. doi: 10.3389/fmed.2026.1687055 (PMC12995620; doi:10.3389/fmed.2026.1687055)
Supplement: Supplementary file 4 [file Table_4.docx]

Supplementary Material

**Table S4. Summary Table of Hyperparameters for 9 Machine Learning Models (Binary Classification - Hernia Prediction**

| Model Name (Engine) | Hyperparameter Search Space (Including Fixed Parameters) | Final Selected Hyperparameters (Selection Criterion) |
| --- | --- | --- |
| Decision Tree (rpart) | Tunable parameters:  tree_depth: [3,7] (integer)  min_n: [5,10] (integer)  cost_complexity: 10^(-6,-1) (log scale)  Fixedparameters:set_args(model=TRUE)  Tuning method: grid_random(size=5) (random grid search) | Optimal combination selected based on ROC-AUC + one-standard-error rule (select_by_one_std_err):  tree_depth: Optimal tree depth  min_n: Optimal minimum number of samples per node  cost_complexity: Optimal complexity penalty value |
| Random Forest (randomForest) | Tunable parameters:  mtry: [2,10] (integer, number of randomly selected features per tree)  trees: [200,500] (integer, number of trees)  min_n: [20,50] (integer, minimum number of samples per node)  Fixed parameters:  set_args(importance = T) (calculate variable importance)  Tuning method: grid_regular(levels=c(2,2,2)) (regular grid search) | Optimal combination selected based on ROC-AUC maximization:  mtry: Optimal number of feature samples  trees: Optimal number of trees  min_n: Optimal minimum number of samples per node |
| XGBoost (xgboost) | Tunable parameters:  mtry: [2,8] (integer)  min_n: [5,20] (integer)  tree_depth: [1,3] (integer)  learn_rate: 10^(-3,-1) (log scale, learning rate)  loss_reduction: 10^(-3,0) (log scale, loss reduction)  sample_prop: [0.8,1] (sample sampling proportion)  Fixed parameters:  trees=1000, stop_iter=25, validation=0.2  Tuning method: grid_random(size=5) (random grid search) | Optimal combination selected based on ROC-AUC maximization:  Optimal values for mtry, min_n, tree_depth, learn_rate, loss_reduction, and sample_prop |
| Elastic Net (glmnet) | Tunable parameters:  mixture: [0,1] (0 = Ridge Regression, 1 = LASSO, 0~1 = Elastic Net)  penalty: 10^(-5,0) (log scale, penalty coefficient)  Fixed parameters:  Binary classification via logistic regression, with standardization and centering preprocessing  Tuning method: grid_regular(levels=c(5,10)) (regular grid search) | Optimal combination selected based on ROC-AUC + one-standard-error rule:  mixture: Optimal penalty mixing coefficient  penalty: Optimal penalty coefficient |
| Regularized Support Vector Machine (SVM)<br>▫️RBF Kernel (main model)<br>▫️Linear Kernel<br>▫️Polynomial Kernel (kernlab) | RBF Kernel (main model)  Tunable parameters:  cost: 10^(-5,5) (log scale, penalty coefficient)  rbf_sigma: 10^(-4,-1) (log scale, kernel width)  Preprocessing: Centering and standardization  Tuning method: grid_regular(levels=c(2,3))  Linear Kernel  Tunable parameter:  cost: 10^(-5,5)  Tuning method: grid_regular(levels=5)  Polynomial Kernel  Tunable parameters:  cost: 10^(-5,5)  degree: Default integer (polynomial degree)  scale_factor: 10^(-5,-1) | Optimal combination selected based on ROC-AUC maximization:  RBF Kernel: Optimal cost + optimal rbf_sigma  Linear Kernel: Optimal cost  Polynomial Kernel: Optimal cost + degree + scale_factor |
| Multi-Layer Perceptron (MLP, nnet) | Tunable parameters:  hidden_units: [15,24] (integer, number of hidden layer neurons)  penalty: 10^(-3,0) (log scale, regularization penalty)  epochs: [50,150] (integer, number of training epochs)  Fixed parameters:  set_args(MaxNWts = 5000) (maximum number of weights)  Preprocessing: Feature normalization via step_range  Tuning method: grid_regular(levels=2) (regular grid search) | Optimal combination selected based on ROC-AUC maximization:  hidden_units: Optimal number of hidden layer neurons  penalty: Optimal regularization coefficient  epochs: Optimal number of training epochs |
| Logistic Regression (glm) | No tunable hyperparameters  Fixed parameters:  Standard binary logistic regression fitting without regularization or penalty terms  No hyperparameter tuning step; direct fitting on the training set | No hyperparameters  Standard logistic regression model that only outputs regression coefficients with no tuning parameters |
| LightGBM (lightgbm, bonsai) | Tunable parameters:  tree_depth: [1,3] (integer)  trees: [100,500] (integer)  learn_rate: 10^(-3,-1) (log scale)  mtry: [2,8] (integer)  min_n: [5,10] (integer)  loss_reduction: 10^(-3,0) (log scale)  Tuning method (choose one):  grid_random(size=5) (random grid search)  tune_bayes (Bayesian optimization, initial=10, iter=30, no_improve=5) | Optimal combination selected based on ROC-AUC maximization:  Optimal values for tree_depth, trees, learn_rate, mtry, min_n, and loss_reduction |
| K-Nearest Neighbors (KNN, kknn) | Tunable parameter:  neighbors: [3,11] (integer, number of nearest neighbors k)  Fixed parameters:  weight_func = "rectangular" (equal weighting)  dist_power = 2 (Euclidean distance)  Tuning method: grid_regular(levels=5) (regular grid search) | Optimal value selected based on ROC-AUC maximization:  neighbors: Optimal number of nearest neighbors k |

Core selection criterion: select_best(metric = "roc_auc") (maximizing ROC-AUC); for decision trees, select_by_one_std_err is used (balancing performance and model simplicity).

Note: All models are built based on the tidymodels framework. Hyperparameter tuning is performed using 5-fold cross-validation, with ROC-AUC as the core metric for optimal hyperparameter selection. Values in range denote parameter intervals, where logarithmic ranges are marked as 10^(x,y) and integer ranges represent continuous integers. All models adopt consistent data preprocessing logic (factor conversion, dummy variable creation, missing value deletion).
