# Supplementary figures and images for: Machine learning-based prediction of hernia risk in peritoneal dialysis patients: a comparative study of models and SHAP-driven interpretability analysis
Source: Front Med (Lausanne). 2026 Mar 4;13:1687055. doi: 10.3389/fmed.2026.1687055 (PMC12995620; doi:10.3389/fmed.2026.1687055)

Supplementary Material

**
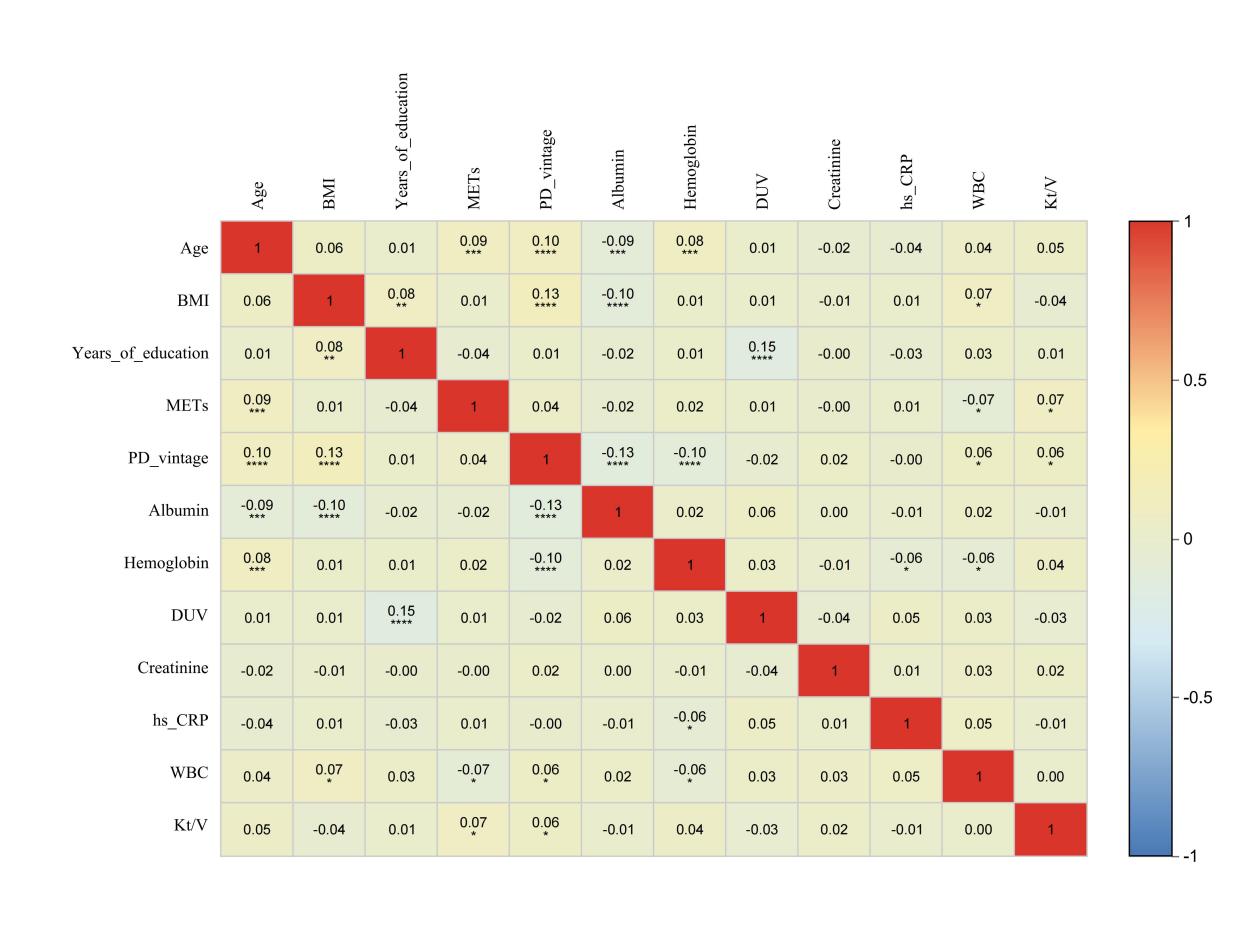
**

Figure S1.Heatmap of Continuous Variable Correlations

Supplement: Supplementary file 5 [file Supplementary_file_1.docx]
